# Knowledge and practices of immediate newborn care among midwives in selected health care facilities in Ekiti State, Nigeria

**DOI:** 10.11604/pamj.2020.37.263.24628

**Published:** 2020-11-24

**Authors:** Deborah Tolulope Esan, Opeyemi Adeniyi Adedeji, Cecilia Bukola Bello, Modupe Christianah Omolafe

**Affiliations:** 1 Department of Nursing Science, College of Medicine and Health Sciences, Afe Babalola University, Ado-Ekiti, Nigeria,; 2 Obstetrics and Gynecology Department, University College Hospital, Ibadan, Nigeria

**Keywords:** Immediate newborn care, midwives, knowledge, practices, neonatal mortality, Nigeria

## Abstract

**Introduction:**

almost 99% of neonatal deaths occur in developing countries and these deaths can be prevented by the presence of a skilled birth attendant during labour. This study therefore assessed the knowledge and practices of midwives towards immediate newborn care.

**Methods:**

this study employed a descriptive cross-sectional design. Eighty nine midwives were selected using purposive sampling technique from the two health facilities. Questionnaire was adapted from helping babies breathe manual. Data collected were analyzed with SPSS version 25 and the results were presented using descriptive statistics.

**Results:**

the mean age of the respondents was 33.20 years ± 8.07. More than half of the midwives (56.2%) had a good knowledge on immediate newborn care. About 62.9% had good practices of immediate new born care, though some still carry out some obsolete practices like routine suctioning of the airway of newborns, immediate cleaning/removal of the vernix caseosa with olive oil and immediate cord clamping.

**Conclusion:**

a bit above average of the midwives had good knowledge about immediate newborn care and some of the midwives still carry out obsolete practices that has been judge as non-beneficial and are not in line with recommendations of World Health Organization/UNICEF on immediate newborn care. This study therefore recommends that midwives undergo training and retraining on immediate newborn care and there should be increased awareness and education on recent up to date practices of immediate newborn care.

## Introduction

Newborn immediate care is the care given to the neonate after birth by qualified personnel in the delivery room [1]. The neonatal and postnatal period, which is the first 28 days and weeks after childbirth is very critical to the survival of both neonate and mother. During this period, major changes occur which determine the well-being of both mothers and their babies [2]. Lack of adequate care could result in significant ill health and even death during this period [2]. During this period most maternal and infant deaths occur, most especially in developing countries [2].

For a child to develop properly and to reach its maximum potential in the future, the care given at birth (i.e. immediate newborn care) is of immense importance [3], which is influenced by newborn care and maternal care services received at health facilities at the time of birth and home care practices of mothers. Causes of neonatal deaths in Nigeria and globally are mostly as a result of infections, prematurity and birth asphyxia [4]. The presence of a skilled professional/birth attendants in labour and delivery can reduce neonatal deaths drastically.

Essential care of the normal newborn with the aim of preventing illnesses, providing extra care for low birth weight babies and quality emergency care for the sick newborn [5], is paramount to newborn survival. The health of a newborn depends on the care provided by the caregivers available during delivery either at home or health facilities. The treatment/care given to the newborn immediately after birth and in both early and late neonatal periods is crucial to its survival. Active participation/involvement of every member of the health team particularly midwives is paramount in providing/given appropriate care to neonates at birth. Simple cost effective interventions such as hygienic cord care and early and exclusive breastfeeding helps in prevention of infection and promote child growth respectively [5]. However, the care needed by newborns may not be appropriately availed to them by their caregivers due to various factors including lack of adequate knowledge, socio cultural, economic and demographic factors affecting mothers as well as care givers.

Neonatal mortality in Nigeria as of today is 36 per 1000 live births and infant mortality rate is 69.8 per 1000 live births which accounts to 20 per cent of child deaths in sub-Saharan Africa still occur in Nigeria. Adebayo *et al*. (2014) [6], attributed neonatal deaths to “endogenous factors” in the newborn, “quality of antenatal care” received by the mother and whether assistance was given “during delivery” and “quality post-partum care”. Knowledge and understanding of midwives on evidence based practice is crucial in reducing neonatal deaths [7]. This research work is therefore aimed at assessing knowledge and practices related to immediate newborn care among midwives in selected health centres in Ado Ekiti Local Government Area, Ekiti State, Nigeria.

## Methods

A cross-sectional descriptive design was used to determine the knowledge and practices of midwives towards immediate newborn care. The study was carried out at Comprehensive Health Center, Okesa, Ado-Ekiti and Ekiti State University Teaching Hospital, Ado-Ekiti, Ekiti State, South West region of Nigeria. Comprehensive Health Center Okesa is a health center located in the core center of Ado-Ekiti, the state capital city of Ekiti State. It was established with the aim of reducing maternal and child mortality at grass root level. The health center comprises of the various units; antenatal clinic, labor ward, post-natal ward, pharmacy and the laboratories. Personnel employed at the health centre include, nurses, 7 midwives, 37 community health extension workers (CHEW) and health/nurse assistant. The services rendered at this health facility includes, antenatal clinic, delivery services, circumcision, family planning, treatment of childhood diseases (for under-five children) and immunization services. Ekiti State University Teaching Hospital, Ado-Ekiti (EKSUTH) is a tertiary health institution and a referral center in Ado-Ekiti, Ekiti State. Personnel include: nurses, midwives, community health extension workers (CHEW) and health/nurse assistant. The obstetrics and gynecology section of the hospital has facilities such as, antenatal ward, obstetrics and gynecology ward, postnatal ward, special care babies unit and labor wards, all referred to as the maternity complex of the hospital. The maternity complex has a total number of 65 midwives, 15 midwives working in the post-natal ward, 18 midwives in the antenatal and gynecology ward and 15 midwives in the special care babies unit, 14 midwives in the labor ward and 3 midwives in the theatre.

The target populations for this study are all midwives that work in the maternity complex in Ekiti State University Teaching Hospital and Comprehensive Health Centre, Ado-Ekiti. Sample size was estimated with Taro Yamane [8] formula, which is:

$$n=\frac{N}{1+N(e^{2})}$$

where: n is sample size required, N is the population size and e is the sampling error (0.05 acceptable error). The N for this study is 102 (37 midwives in Comprehensive Health Center and 65 midwives in EKSUTH). Using this formula, the sample size yielded 81 participants. Adding an attrition rate of 10% to cater for non-responses, yielding a total of 89 respondents required for the study.

A purposive sampling technique was used in this study because the study focused only on midwives in the study areas in Ado Ekiti, Ekiti State. The inclusion criteria for this study included. All midwives that work in the maternity complex of the health facility and willingness to participate in the study and nurses and midwives that are not working in the maternity complex of the hospital and were unwilling to participate were excluded from the study. The research instrument for data collection in this study was a well-structured adapted questionnaire. The items of the questionnaire were adapted “Helping Babies Breathe manual” by projectc.u.r.e and also from published articles [9,10]. Items on the questionnaire comprised of 3 sections: section A included questions on the demographic profile of respondents; section B contained questions assessing the knowledge of midwives on immediate new born care; and section C included questions designed to assess the practices of midwives on immediate newborn care. Midwives working at the maternity complex of the hospitals were asked to participate in the study. Each participant was informed about the purpose of the study. The questionnaires were administered to the respondents by the researcher during the shift periods at their workplaces.

Data was presented, organized and summarized numerically using descriptive statistics of frequencies, percentages, pie and bar charts. Data analysis was done with the aid of Statistical package for Social Sciences (SPSS) version 25.0 to generate figures and graphs. The data collected was analyzed with the use of tables, frequency charts and percentages, which was interpreted and conclusions were drawn as appropriate. Regarding questions to assess midwives knowledge on immediate newborn care, respondents were graded based on 13 items on the questionnaire. Each correct score was given 1 mark while each incorrect score was given 0 mark making a total of 13 marks. Respondents that scores between 0-4 were said to have “poor knowledge” while those that score between 5 and 8 were said to have “fair knowledge” and those that score between 9 and above were said to have a “good knowledge”. Regarding practice of midwives on immediate newborn care, composite score was graded on a 2 point scale based on 12 items on the questionnaire. Each correct answer was scored 1 and incorrect response was scored 0, with a total aggregate of 12 scores. Respondents scores between 0-6 were said to have “poor practice” and scores between 6 -12 were said to have “good practice”.

Ethical approval was obtained from the Ethics and Research Committee of Ekiti State University Teaching Hospital, Ado-Ekiti. Prior to the commencement of data collection, informed consent was obtained from each respondent.

## Results

Total of 89 questionnaires were distributed and returned, after sorting out the questionnaires. All were found to be complete enough for analysis. Data collected were analyzed based on the responses received from the respondents. The data were collated and arranged in line with set objectives. Table 1 revealed a descriptive socio-demographic characteristic of respondents. From the result, more than two third (77.5%) of the respondents were married, the mean age of the respondents were 33.20 ± 8.07 while majority (41.6%) were within the age range of 30-39 years and (84.3%) of respondents hailed from Yoruba ethnicity. 96.5% of respondents were christians, about 59.5% percent had a bachelor degree in nursing sciences. Likewise, 54% had less than five years working population in the maternity unit, 50.6% of the respondents were working in labour ward while 60.7% of the respondents had worked for one to five years within their working institution.

**Table 1:** socio-demographic characteristics of respondents

Variables	Frequency (N=89)	Percentage (%)
**Age**		
20-29	35	39.3
30-39	37	41.6
40-49	12	13.5
Above 49	5	5.6
Mean age=33.20 ± 8.07		
**Tribe**		
Yoruba	75	84.3
Hausa	4	4.5
Igbo	6	6.7
Others	4	4.5
**Religion**		
Christian	77	96.5
Islam	12	13.5
**Marital status**		
Single	16	18.0
Married	69	77.5
Co-habiting	2	2.2
Divorced	1	1.1
Widowed	1	1.1
**Highest nursing education**		
Diploma	34	38.2
Degree	53	59.5
MSc	2	2.2
**How long have you been working in the maternity unit**		
Less than 5 years	48	54.0
5-10 years	31	34.8
More than 1 year	10	11.2
**What section of the maternity unit are you working**		
Labor ward	45	50.6
SCBU	13	14.6
Post-natal ward	15	16.5
Antenatal ward	13	14.6
Theatre	3	3.4
**How many years have you worked in your present institution**		
Less than 5 years	54	60.7
5-10 years	31	34.8
More than 10 years	4	4.5

Table 2 revealed the respondents knowledge on immediate newborn care. About 65% of the respondents were of the opinion that immediately after drying the baby skin to skin care begins while 58.4% of the respondents agreed that at least six hours after birth the baby should have its first birth. However, 79.8% of the respondents were of the notion that the newly born baby should be wiped with clean cloth during the first hour after birth. More so 60.7% and 71.9% of the respondents both agreed after three hours, 8-12 times in a day likewise within 90 minutes after birth a baby need to be feed and weighed respectively. More than half of the respondents (52.8%) revealed that initial eye treatment should be given within 90 minutes after birth. Inherently, 64% of the respondents also hold the view that eye should medicate to prevent eye infection inside the lower eyelid. Likewise, 66.3% of the respondents were of the opinion that every 15 minutes the baby should be observed for breathing problems. Furthermore, majority of the respondents (91%) used to wrap in a clean, dry blanket or cloth to keep warm after skin-skin care.

**Table 2:** knowledge of immediate newborn care

Variables	Frequency	Percentage
**When should skin-to-skin care begin**		
After delivery of the placenta	26	29.2
Immediately after drying the baby following birth	58	65.2
After being shown to the relatives	5	5.6
**When should a baby be first bathed**		
As soon as the baby is born	12	13.5
At least 6 hours following birth	52	58.4
As soon as the baby has a normal temperature	25	28.1
**During the first hour after birth, how should the meconium on the baby's skin be removed**		
Scrubbed away in a bath of warm water	13	14.6
Wiped away with clean cloth	71	79.8
Scrubbed with a cloth containing alcohol	5	5.6
**During the first hour should skin-skin care be interrupted**		
Briefly for essential care only	76	85.4
When showing the baby to the relatives	7	7.9
To give first bath	6	6.7
**How often should healthy babies feed**		
Ever one hour	30	34.7
About every 3 hours, 8-12 times in a day	54	60.7
Every 6 hours	5	5.6
**How soon after birth should the baby be weighed**		
Sometime during the first day	11	12.4
Any time before the mother and baby leave the birth facility	14	15.7
Within 90-minutes after birth	64	71.9
**When should initial eye treatment be given**		
At the end of the first day	20	22.5
Within 90 minutes after birth	47	52.8
After the first bath	22	24.7
**In what part of the eye should medication to prevent eye infection be given**		
Inside the upper eyelid	5	5.6
Inside the lower eyelid	57	64.0
Inside the cornea of the eye only	27	30.4
**During the first hour how often should the baby be observed for breathing problems**		
Once in the hour	3	3.4
Every 30 minutes	27	30.3
Every 15 minutes	59	66.3
**How should a bay be kept warm after skin-skin care**		
Bathing in warm water	6	6.7
Wrapping in a clean, dry blanket or cloth	81	91.0
Exposing the baby to direct sunlight	2	2.3

**Figure 1:**
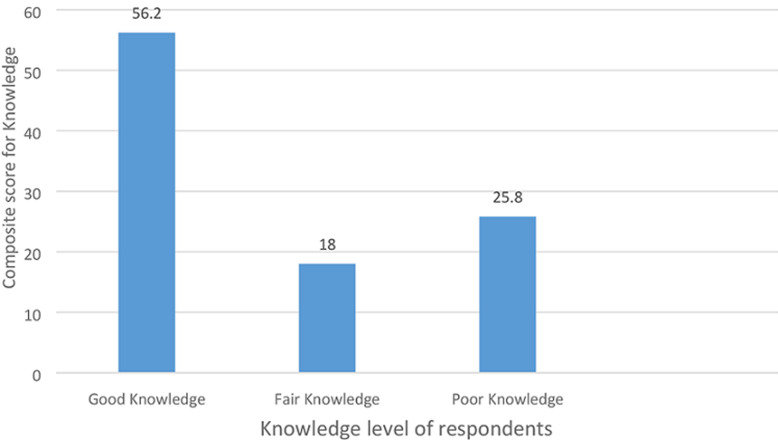
summary of knowledge level of midwives on immediate newborn care

The composite of the knowledge score of respondents on immediate newborn care revealed that 56% of the midwives have good knowledge, 18% had fair knowledge and 25.8% had poor knowledge (Figure 1). Table 3 revealed the immediate newborn care practices of midwives. A substantial number of the respondents immediately after delivery wipe the eye of the new born against 59.6% of the respondents immediately also dry the body of the new born. However, 64% of the respondents also immediately clamp and cut the cord of a new born while 73% of the respondents initiate breastfeeding within 30 minutes after birth. Inherently, 58.4% of the respondents cleared the airway of the new born routinely, while, majority (82%) of the respondents clean the vernix of the new born immediately. Likewise, 77.5% of the respondents begin to do ventilation if the baby does not cry after birth while 78.7% of the respondents also place the baby on the mothers´ abdomen. Majority of the respondents (92.2%) also measure the weight, length and head circumference of the new born while 74.2% of the respondents also agreed that they placed the baby´s identification band on the ankle or wrist. The composite score of practice of midwives on immediate newborn care was grouped into two (good practice and poor practice). Findings/result revealed that 62.9% of the midwives had good practice and 37.1% had poor practice.

**Table 3:** immediate newborn practices

Variables	Frequency	Percentage
**When do you wipe the eyes of the new born**		
Immediately after delivery of the baby's head	79	88.8
After delivery of the baby's head and limbs	8	9.0
After the first bath	2	2.2
**When do you dry the body of the new born**		
Immediately the baby is born	53	59.6
After 1-3 minutes of birth	24	27.0
After 5 minutes of birth	12	13.5
**When do you clamp and cut the cut of a new born**		
Immediately the baby is born	57	64.0
After 1-3 minutes of birth	22	24.7
After 5 minutes of birth	10	11.2
**When do you initiate breastfeeding**		
Within 30 minutes after birth	65	73.0
Within one hour after birth	19	21.3
After one hour of birth	5	5.6
**Do you clear the airway of every new born**		
Yes	52	58.4
No	14	15.7
If needed	23	25.9
**Do you clean the vernix (the white creamy substance found on the baby's skin) off every new born immediately after birth**		
Yes	73	82.0
No	7	7.9
If needed	9	10.1
**What do you do if the baby does not cry at birth**		
Begin ventilation	69	77.5
Slap the baby's back	17	19.1
Call the doctor	3	3.4
**Do you place the baby on the mother's abdomen**		
Yes	70	78.7
No	10	11.2
If needed	9	10.1
**Do you measure the weight, length and head circumference of the newborn**		
Yes	82	92.2
No	5	5.6
If needed	2	2.2
**Do you place the baby's identification band on the ankle or wrist**		
Yes	66	74.2
No	9	10.1
If needed	14	15.7

## Discussion

This study found that the proportion of midwives with good knowledge of immediate newborn care was 56 percent, which is almost close to North-east Ethiopia findings (53.8%) [11], Addis Ababa (51%) [12] and Bahir Dar city (56%) [10]. This finding, by contrast, is less than the Jimma studies (66.4%) [13], eastern Tigray (75%) [14], North-western Tigray (64.8%) [15] and Pune city 72% [16].

This knowledge level seen in this study is not satisfactory, as the importance of good knowledge regarding immediate newborn care of skilled birth attendants cannot be undermined in reducing neonatal death in Nigeria. This was noted by Monebenimp *et al*. 2012 [17], who reported an increase in birth death may be linked to a lack of knowledge of neonatal birth resuscitation. The study revealed that majority (62.9%) had good practice of immediate new born care. This finding is in tandem with the findings in Northeast Ethiopia 62.7% [11], Bahir Dar city 59.7% [10] and North-western zone of Tigray 59.8% [15]. In comparison, our result is significantly higher than the analysis in the Tigray region's central zone of public health facilities [9], but lower than in Jimma (68.3%) [14], Eastern zone of Tigray (73%) [15], Addis Ababa (81%) [12] and Pune city (98%) [16]. Abdu *et al*. 2019 [11] indicated that those differences may be due to the disparity in the instruments and criteria used to assess practice of respondents.

Most of the respondents immediately after delivery wipe the eye of the new born while approximately 60% of the study subject, immediately also dry the body of the new born and initiate breastfeeding within 30 minutes after birth. The care midwives provide at birth gives a vital support in avoiding complications and ensuring survival at the time of birth. Similarly, Saaka *et al*. (2014) [18], noted that skilled care during labour and childbirth with prompt complication management alone can lead to about 50% reduction in newborn deaths and 45% of intra-partum deaths. Seventy-five percent of current neonatal deaths can be avoided with appropriate postnatal treatment [18].

Although more than half (57%) of these midwives practice immediate clamping of the cord this is contrary to the recommendation by the resuscitation council (2011) recommends that cord clamping should be delayed for at least five minutes after birth as it allows oxygenated blood to be transferred from the placenta to the baby. Majority of the midwives (73%) clean the vernix of the newborn immediately after birth and this contradicts the current recommendation by WHO, (2014) [2] to delay cleaning of the newborn´s vernix for at least 6 hours and may be up to 24 hours as it has beneficial effects to the new born like retention of moisture (aids thermoregulation in newborn), antimicrobial effects and it also helps baby to latch on as the scent can trigger neural connection of baby´s brain needed for breastfeeding therefore this practice of cleaning of vernix cleansing should be discouraged among midwives as it does more harm than good for the baby. More than half of the midwives (52%) clear the airway of the newborn after birth which is not in line with the current WHO (2014) [2] recommendation to discourage routine suctioning of babies who start breathing on their own and only to suction new born if they have secretions clearly blocking their nose and mouth.

## Conclusion

Knowledge level of midwives in this study on immediate newborn care is fair and the practice is also not good enough considering the current neonatal mortality rate in Nigeria. It therefore brings attention to the need for further awareness and education on the current practices of immediate new born care for midwives/birth attendants in Ekiti State, Nigeria, through emphasizing knowledge, positive and good practices, as doing this can reduce neonatal mortality rate in Nigeria. It is therefore recommended that the issue of knowledge transfer and translation of knowledge into practice should be done through pre-service and in-service education, update courses and workshops, which could serve as a form of empowerment for professional nurse midwives, who are supposed to be abreast of current trend and practice, in order to ensure child survival and that every child is given an opportunity to live.

**Implications of findings for practice:** adequate knowledge and good/standard practice of midwives and other health care professionals on immediate care of newborns in Nigeria can drastically reduce neonatal mortality. Likewise the curbing of complications and early detection of complications can be achieved with standard practice given the fact that babies cannot complain or express directly the pains they are going through; high level of observation and knowledge retention and application are required in detecting these complications or issues faced by newborn, thus improving the current neonatal mortality statistics.

### What is known about this topic

- The presence of a skilled professional/birth attendants in labour and delivery can reduce neonatal deaths drastically;
- Essential care of the normal newborn with the aim of preventing illnesses, providing extra care for low birth weight babies and quality emergency care for the sick newborn is paramount to newborn survival;
- For a child to develop properly and to reach its maximum potential in the future, the care given at birth (i.e. immediate newborn care) is of immense importance, which is influenced by newborn care and maternal care services received at health facilities.

### What this study adds

- The study helps in identifying training needs of midwives in order to ensure quality care is given to pregnant women during delivery;
- It further brings attention to the need for further awareness and education on the current practices of immediate new born care for midwives/birth attendants in Ekiti State, Nigeria, as doing this can reduce neonatal mortality rate in Nigeria.
